# USP22‐mediated deubiquitination of PTEN inhibits pancreatic cancer progression by inducing p21 expression

**DOI:** 10.1002/1878-0261.13137

**Published:** 2021-11-16

**Authors:** Dianyun Ren, Yan Sun, Dan Li, Heshui Wu, Xin Jin

**Affiliations:** ^1^ Department of Pancreatic Surgery Union Hospital Tongji Medical College Huazhong University of Science and Technology Wuhan China; ^2^ Sino‐German Laboratory of Personalized Medicine for Pancreatic Cancer Union Hospital Tongji Medical College Huazhong University of Science and Technology Wuhan China; ^3^ Cardiovascular Medicine Department Union Hospital Tongji Medical College Huazhong University of Science and Technology Wuhan China; ^4^ Department of Urology The Second Xiangya Hospital Central South University Changsha China

**Keywords:** ANKHD1, p21, pancreatic cancer, PTEN, USP22

## Abstract

Phosphatase and tensin homolog deleted on chromosome ten (PTEN) is a dual lipid and protein phosphatase. Multiple mechanisms contributing to the regulation of PTEN levels have been identified thus far, including post‐translational modifications, epigenetic mechanisms, and transcriptional mechanisms. In the present study, we identified ubiquitin‐specific peptidase 22 (USP22) as a novel deubiquitination‐modifying enzyme of PTEN. Furthermore, by inducing deubiquitination and inhibiting the degradation of PTEN, USP22 could induce cyclin‐dependent kinase inhibitor 1A (CDKN1A, also symboled as p21) expression in pancreatic cancer. Besides, MDM2 proto‐oncogene (MDM2) inhibitor enhanced the antipancreatic cancer effects of USP22 overexpression. In addition to its regulation of MDM2‐tumor protein p53 (p53) signaling, we found that PTEN could induce p21 expression by interacting with ankyrin repeat and KH domain containing 1 (ANKHD1) and inhibiting ANKHD1 binding to the p21 promoter. Taken together, our results indicate that ANKHD1 and MDM2 might be novel therapeutic targets in pancreatic cancer.

## Graphical abstract

The expression of PTEN was induced by ubiquitin‐specific peptidase 22 (USP22) in pancreatic cancer, which depended on the deubiquitinating function of USP22. PTEN induced the expression of p21 via MDM2‐p53 pathway and via interacting with ANKHD1. Taken together, USP22 could also induce the expression of p21 via deubiquitinating and upregulating the expression of PTEN in pancreatic cancer.

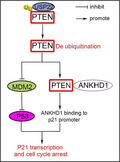

**Abbreviations**
ANKHD1: ankyrin repeat and KH domain containing 1
CDKN1A: cyclin‐dependent kinase inhibitor 1A, also symboled as p21
KEGG: Kyoto Encyclopedia of Genes and Genomes
LC‐MS/MS: liquid chromatography‐tandem mass spectrometry/mass spectrometry
MDM2: MDM2 proto‐oncogene
p53: tumor protein p53
PDAC: pancreatic ductal adenocarcinoma
PI3K: phosphatidylinositol 3‐kinase
PIP2: plasma membrane intrinsic protein 2
PIP3: plasma membrane intrinsic protein 3
PTEN: phosphatase and tensin homolog deleted on chromosome ten
PTM: post‐translational modification
rhUSP22: recombinant human USP22
USP22: ubiquitin‐specific peptidase 22

## 1 Introduction

Pancreatic ductal adenocarcinoma (PDAC) is a highly lethal human cancer with an overall 5‐year survival rate of 7% [1]. PDAC is expected to become the second leading cause of cancer‐related deaths by the year 2030 [2]. Approximately 80% of patients with pancreatic cancer lose the opportunity for surgical resection [3]. Although there has been marginal progress regarding chemotherapy, pancreatic cancer continues to exhibit poor prognosis [3]. Therefore, novel therapeutic strategies are urgently needed for PDAC treatment.

Phosphatase and tensin homolog deleted on chromosome ten (PTEN) is a dual lipid and protein phosphatase. The most extensively characterized biochemical function of PTEN comprises its lipid phosphatase activity. PTEN antagonizes phosphatidylinositol 3‐kinase (PI3K) signaling by hydrolyzing plasma membrane intrinsic protein 3 (PIP3) to generate plasma membrane intrinsic protein 2 (PIP2), thereby inhibiting cell survival, growth, migration, and stem cell self‐renewal [4, 5]. PTEN has also been reported to exhibit protein phosphatase activity, which is responsible for some of its biological effects, including cell migration inhibition and cell cycle arrest [6]. The tumor suppressor function of PTEN involves negative regulation of the PI3K/PTEN/Akt signaling pathway that governs cellular functions, including survival, proliferation, energy metabolism, and cellular architecture [7]. Moreover, PTEN may regulate cell cycle progression and cell invasion by means of its protein phosphatase activity; chromosomal stability may be regulated through its nuclear localization [8, 9]. However, further research is needed regarding potential other mechanisms by which PTEN may inhibit the progression of pancreatic cancer.

Loss of PTEN function occurs in a wide spectrum of human cancers through various mechanisms, including mutations, deletions, transcriptional silencing, or protein instability [10]. Multiple mechanisms contributing to the regulation of PTEN levels have been identified thus far, including post‐translational modifications (PTMs) [11], epigenetic mechanisms [12], and transcriptional mechanisms [13]. Furthermore, PTEN is subject to a range of regulatory mechanisms (e.g., PTMs, PTEN‐interacting proteins, dimerization, and secretion) that ultimately govern its protein levels, activity, and function [14]. Recently, the inhibition of PTEN expression has been shown to promote cell proliferation [15], cancer metastasis [16], invasion [17], tumorigenesis and progression [18], and drug resistance [19] in pancreatic cancer. Therefore, mechanistic exploration is needed regarding the regulation of PTEN expression in pancreatic cancer.

In the present study, we demonstrated that restoring the expression of PTEN significantly inhibited the progression of pancreatic cancer. Furthermore, upregulating the expression of PTEN led to p21 expression in pancreatic cancer via both tumor protein p53 (p53)‐dependent and p53‐independent signaling pathways. In addition, ubiquitin‐specific peptidase 22 (USP22) induced p21 expression by deubiquitinating PTEN in pancreatic cancer. Therefore, our study indicated that both PTEN and USP22 could be novel therapeutic targets for pancreatic cancer.

## 2 Materials and methods

### 2.1 Cell culture

The pancreatic cancer cell lines SW 1990 and HPAC were purchased from ATCC (Washington, DC, USA). SW 1990 cells were cultured in L15 medium (Gibco, Grand Island, NY, USA) supplemented with 10% FBS and 1% penicillin/streptomycin at 37 °C in a 5% CO_2_ incubator. HPAC cells were cultured in Dulbecco’s modified Eagle’s medium (Gibco) supplemented with 10% FBS and 1% penicillin/streptomycin at 37 °C in a 5% CO_2_ incubator.

### 2.2 Chemicals

The reagent MG132 (HY‐13259, Med Chem Express, Shanghai, China) was used at 10 μm. The reagent Nutlin‐3a (HY‐10029, Med Chem Express) was used at 10 μm *in vitro* (IC_50_ = 17.75 and 25.67 µm for SW 1990 and HPAC, respectively, Fig. S1) and 100 mg·kg^−1^ *in vivo*. Cycloheximide (HY‐12320, Med Chem Express) was used at 1 μm.

### 2.3 Immunoprecipitation, western blotting, and antibodies

The methods for immunoprecipitation and western blotting assays have been described previously [20]. Cell lysates were prepared in radioimmunoprecipitation assay (RIPA) buffer (Thermo Fisher Scientific, Waltham, MA, USA) supplemented with protease inhibitor cocktail (Sigma‐Aldrich, St. Louis, MO, USA) and Halt Phosphatase Inhibitor Cocktail (Thermo Scientific, Waltham, MA, USA). Primary antibody (0.5–2.0 µg; listed below) was added to the cell lysate, and the sample was incubated on ice overnight. The next day, 20–50 µL of Protein A beads (Cat# 9863, CST, Danvers, MA, USA) was added, and the sample was incubated under rotation at 4 °C for 3 h. The next day, the beads were washed at least six times with lysate buffer on ice and then subjected to western blotting analysis.

Equal amounts of protein were separated by 10% sodium dodecyl sulfate/polyacrylamide gel electrophoresis (SDS/PAGE), transferred onto polyvinylidene fluoride membranes (Millipore, Boston, MA, USA), and incubated with the appropriate antibodies. Protein signals were visualized using the ECL detection reagent (Thermo Fisher Scientific) and ChemiDoc XRS (Bio‐Rad Laboratories, Hercules, CA, USA). GAPDH was used as a loading control.

SGLT2 antibody was purchased from Cell Signaling Technologies (Danvers, MA, USA) (Cat# 14210, WB: 1 : 1000, IP: 1 : 50) and from Proteintech (Wuhan, China) (Cat# 24654‐1‐AP, Immunohistochemistry (IH0C): 1 : 400); GAPDH antibody was purchased from Proteintech (Cat# 10494‐1‐AP, WB: 1 : 3000); PTEN antibody was purchased from Proteintech (Cat# 22034‐1‐AP, WB: 1 : 1000); p21 antibody was purchased from Proteintech (Cat# 10355‐1‐AP, WB: 1 : 1000); p53 antibody was purchased from Proteintech (Cat# 10442‐1‐AP, WB: 1 : 1000); MDM2 proto‐oncogene (MDM2) antibody was purchased from Proteintech (Cat# 19058‐1‐AP, WB: 1 : 1000); ankyrin repeat and KH domain containing 1 (ANKHD1) antibody was purchased from Absin (Beijing, China) (Cat# abs118369, WB: 1 : 1000); and USP22 antibody was purchased from Proteintech (Cat# 55110‐1‐AP, WB: 1 : 1000, IP: 1 : 50).

### 2.4 Liquid chromatography‐tandem mass spectrometry/mass spectrometry analysis

293T cells transfected with a Flag‐PTEN‐expressing plasmid were used to identify novel PTEN‐binding proteins. PTEN was immunoprecipitated using an anti‐PTEN antibody and protein A+G agarose (#P2012, Beyotime, Shanghai, China) at 4 °C. Liquid chromatography‐tandem mass spectrometry/mass spectrometry (LC‐MS/MS) analysis was performed using a Thermo Ultimate 3000 liquid phase combined with Q Exactive Plus high‐resolution mass spectrometry at Shanghai Applied Protein Technology. The data were retrieved using maxquant (v1.6.6) software (Am Klopferspitz, Martinsried, Germany) and the algorithm Andromeda. The reference database comprised the UniProt human proteome reference database. Proteins and peptides with a false discovery rate (FDR) of 1% were selected.

### 2.5 RNA sequencing

The methods for RNA sequencing assay have been described previously [20]. In total, 1 µg of RNA per sample was used as the input material for RNA sequencing (RNA‐seq). Sequencing libraries were generated using the NEBNext Ultra RNA Library Prep Kit for Illumina (NEB, Ipswich, MA, USA), in accordance with the manufacturer's instructions. Clustering of the samples was performed on the cBot Cluster Generation System using the TruSeq PE Cluster Kit v3‐cBot‐HS (Illumina, San Diego, CA, USA), in accordance with the manufacturer’s instructions. After cluster generation, libraries were sequenced on an Illumina NovaSeq platform, and 150‐bp paired‐end reads were generated. featurecounts v1.5.0‐p3 (Bioconductor, TU Dortmund, Germany) was used to count the read numbers mapped to each gene. Differential expression analysis (two biological replicates per condition) was performed using the deseq2 r package (1.16.1) (Bioconductor, TU Dortmund, Germany). The clusterprofiler r package (Bioconductor, TU Dortmund, Germany) was used to test the statistical enrichment of differentially expressed genes (DEGs) in Kyoto Encyclopedia of Genes and Genomes (KEGG) pathways.

### 2.6 Quantitative real‐time PCR (qRT‐PCR)

Total RNA was prepared using TRIzol reagent (Cat# 15596026, Invitrogen, Carlsbad, CA, USA). Total RNA was reverse‐transcribed into first‐strand cDNA using the PrimeScript RT Reagent Kit (Cat# RR047A, Takara, Osaka, Japan). Quantitative PCR assays were carried out with the TB Green Fast qPCR Mix kit (Cat# RR430A, Takara). Results were normalized to the mRNA level of *GAPDH*. Values represent the means of three technical replicates from at least three independent experiments (biological replicates). Primer sequences are shown in Table S1.

### 2.7 RNA interference

Human expression vectors for FLAG‐ITGA2 recombinant proteins were generated using the pcDNA3.1 backbone vector. Lipofectamine 2000 (Invitrogen) diluted in Opti‐MEM medium (Invitrogen) was used to transfect short‐hairpin RNA (shRNA) plasmids and viral packaging plasmids (pVSV‐G and pEXQV) into 293T cells. At 24 h after transfection, the medium was replaced with fresh Dulbecco’s modified Eagle’s medium containing 10% FBS and 1 mm sodium pyruvate. At 48 h after transfection, the culture medium containing viral particles was collected and then added to pancreatic cells along with 12 μg·mL^−1^ of polybrene. At 24 h after infection, the infected cells were selected with 10 μg·mL^−1^ of puromycin. shRNA sequences are provided in Table S2.

### 2.8 Chromatin immunoprecipitation (ChIP) and ChIP‐qRT‐PCR

ChIP assays were performed using an EZ‐ChIP kit (Millipore), in accordance with the manufacturer’s instructions. Immunoprecipitation was performed using the appropriate antibodies and a mouse IgG antibody as a negative control. The primers were designed according to the promoter sequences of the genes of interest. The sequences of the ChIP‐qRT‐PCR primers are shown in Table S3.

### 2.9 Colony formation assay

Pancreatic cancer cells were seeded in six‐well plates (500 cells·well^−1^) and cultured for 10–12 days in complete growth medium containing 10% FBS, which was replaced at 3‐day intervals. Two weeks after seeding, colonies were fixed with methanol for 30 min and then stained with a phosphate‐buffered saline (PBS) solution containing 0.1% crystal violet (Sigma‐Aldrich) for 30 min. Colonies were photographed and counted. All experiments were repeated three times.

### 2.10 MTS assay

The viability of cells seeded in 96‐well plates was tested using 3‐(4,5‐dimethylthiazol‐2‐yl)‐5‐(3‐carboxymethoxyphenyl)‐2‐(4‐sulfophenyl)‐2H‐tetrazolium (MTS) reagent (Cat# ab197010, Abcam, Chicago, IL, USA). MTS reagent (20 µL) was added to each well, and cells were incubated for 3 h. Cell proliferation was assessed at 24‐h intervals after cell transfection. Optical absorbance at 490 nm was assessed on a microplate reader. All experiments were repeated three times.

### 2.11 Cell cycle analysis

The method for cell cycle analysis was referred to the study conducted by Boyan Huang etc. [21]. Cells were washed with precooled PBS after digestion with 0.25% trypsin (Gibco) and fixed with 70% ethanol at 4 °C overnight. Cells were then washed with precooled PBS and collected via centrifugation. Cells were resuspended with 500 μL of PI staining reagent (Beyotime) and incubated for 30 min at 37 °C. Cell cycle was assessed using flow cytometry (BD, Franklin Lakes, NJ, USA), and the data were analyzed with flow jo™ software (BD).

### 2.12 Apoptosis analysis

The method for apoptosis analysis was referred to the study conducted by Boyan Huang etc. [21]. Apoptosis analysis was performed using an Annexin V‐FITC/PI kit (Beyotime). The culture medium was discarded, and the cells were digested with 0.25% trypsin and then washed twice with precooled PBS. Next, the cells were resuspended in 1× binding buffer and 100 μL of cell solution was transferred to a new tube. Five microliters each of the Annexin V‐FITC and PI solutions were added to each cell solution. The cells were gently vortexed and incubated for 15 min at 25 °C in the dark and then mixed with 400 μL of 1× binding buffer per sample. Apoptosis was measured via flow cytometry (BD), and the data were analyzed with flow jo™ software (BD).

### 2.13 Immunohistochemistry

Mouse tumor sections were stained with Ki‐67 antibody (Proteintech, 1 : 10000) to determine cell proliferation. Positively stained color (brown in each case) was selected for quantification of the relative intensity.

### 2.14 Pancreatic cancer xenografts in nude mice

Animal experimental procedures were approved by the Ethics Committee of Tongji Medical College, Huazhong University of Science and Technology (IACUC Number: 2551). Athymic nude (nu/nu) mice (4–5 weeks old, male) were purchased from Vital River (Beijing, China) and fed in a special pathogen‐free animal facility and allowed to eat and drink *ad libitum*. In total, 5 × 10^6^ SW 1990 cells were dispersed in 100 µL PBS and were subcutaneously injected into the left dorsal flank of nude mice. The mice were intratumoral injected with Nutlin‐3a for 3 times on days 1, 4, and 7 at a dose of 100 mg·kg^−1^. Tumor sizes were measured with a digital Vernier caliper at 2‐day intervals for 21 days. Tumor volumes were calculated using the following formula: tumor volume (mm^3^) = (L × W^2^)/2. Mice were sacrificed on day 21 or when tumor volume reached 1000 mm^3^.

### 2.15 Statistical analysis

All experiments were performed at least three times. Parametric data are shown as means ± standard deviations (SDs) and nonparametric data as medians and ranges. Two‐way ANOVA or one‐way ANOVA with Tukey’s multiple comparison test was used for multiple group analysis. Unpaired Student’s *t*‐tests were used to compare data between two groups. Two‐tailed *P*‐values < 0.05 were considered statistically significant. All statistical analyses were performed using graphpad prism 6 software (GraphPad Software, Inc, San Diego, CA, USA).

## 3 Results

### 3.1 USP22 deubiquitinated PTEN and inhibited the degradation of PTEN in pancreatic cancer

**Fig. 1:**
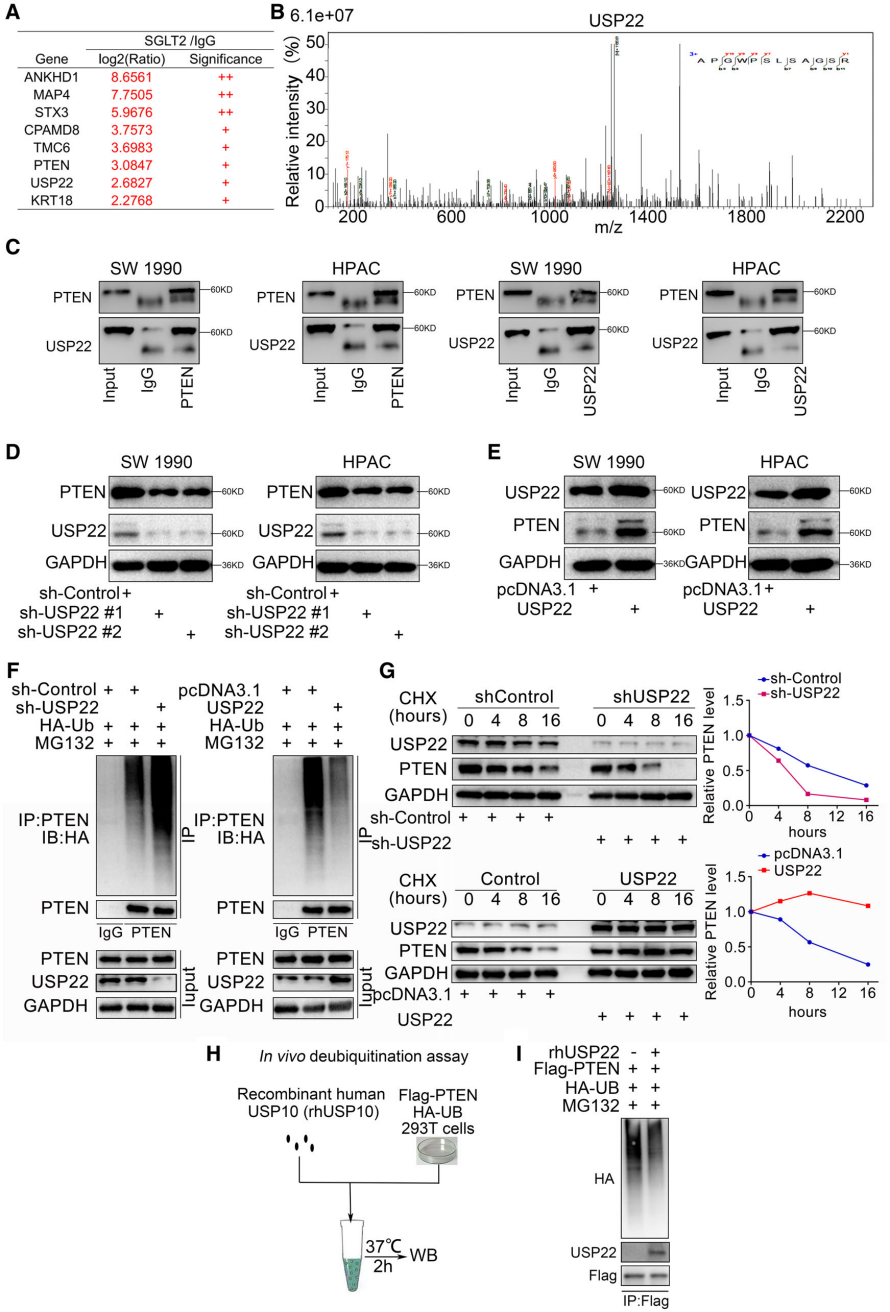
USP22 deubiquitinated PTEN and inhibited the degradation of PTEN in pancreatic cancer. (A,B) LC‐MS/MS identified an interaction between PTEN and USP22 by detecting a peptide of USP22 (*n* = 1 for IgG and PTEN group). (C) Coimmunoprecipitation showed the interaction between PTEN and USP22, which repeated for three replicates. (D,E) Western blot analysis of PTEN expression in SW 1990 and HPAC cells. GAPDH served as an internal reference and repeated for three replicates. (F) Western blot analysis in SW 1990 cells transfected with sh‐USP22 or USP22 plasmid for 48 h for western blot. Cells were treated with MG132 (10 µm) for 8 h before harvested. GAPDH served as an internal reference and repeated for three replicates. (G) Western Blot to show the PTEN expression in SW 1990 and HPAC cells. The cells were treated with cycloheximide for different duration. GAPDH served as an internal reference and repeated for three replicates. (H,I) Flag‐tagged PTEN and HA‐tagged UB plasmids were transfected into 293T cells. Subsequently, polyubiquitinated PTEN from the cell lysate pulled down by anti‐Flag IP resin and incubated with rhUSP22 protein for 2 h at 37 °C *in vitro* (H). Lysates were immunoblotted with indicated antibodies (I). GAPDH served as an internal reference and repeated for three replicates. GAPDH: Glyceraldehyde‐3‐phosphate dehydrogenase; IP: Immunoprecipitation.

PTEN is known to inhibit the PI3K/AKT pathway in cancer cells and the PTM of PTEN is important for its function. Thus, we performed LC‐MS/MS analysis to identify PTEN‐associated proteins in 293T cells transfected with a Flag‐PTEN‐expressing plasmid. We found that PTEN might interact with USP22 (Fig. 1A,B, and S2). Subsequently, the PTEN‐USP22 interaction was confirmed by endogenous immunoprecipitation analysis in both SW1990 and HPAC cells (Fig. 1C). As a de‐ubiquitination‐related molecule, USP22 suppresses cancer progression by inhibiting the degradation of various proteins [22, 23]. Notably, USP22 silencing significantly inhibited the expression of PTEN at the protein level (Fig. 1D), whereas USP22 overexpression induced the expression of PTEN at the protein level (Fig. 1E). Furthermore, MG132 (10 µm) was used to inhibit the degradation but not change the ubiquitination level of PTEN, and ubiquitin immunoprecipitation and half‐life determination assays indicated that USP22 silencing led to the enhancement of PTEN degradation, whereas USP22 overexpression led to the inhibition of PTEN degradation (Fig. 1F,G). To further confirm the deubiquitination activity of USP22 toward PTEN, we utilized a cell‐free system and performed an *in vitro* deubiquitination assay using bacterial‐expressed recombinant human USP22 (rhUSP22). Flag‐PTEN and HA‐UB plasmids were transfected into 293T cells. Subsequently, polyubiquitinated PTEN from the cell lysate pulled down by anti‐Flag IP resin and incubated with rhUSP22 protein for 2 h at 37 °C *in vitro* (Fig. 1H). The results showed that rhUSP22 effectively removed the polyubiquitination from PTEN (Fig. 1I). Therefore, USP22 deubiquitinated PTEN and inhibited the degradation of PTEN; upregulation of USP22 expression could restore PTEN expression in pancreatic cancer.

### 3.2 Restoration of PTEN expression inhibited the progression of pancreatic cancer

**Fig. 2:**
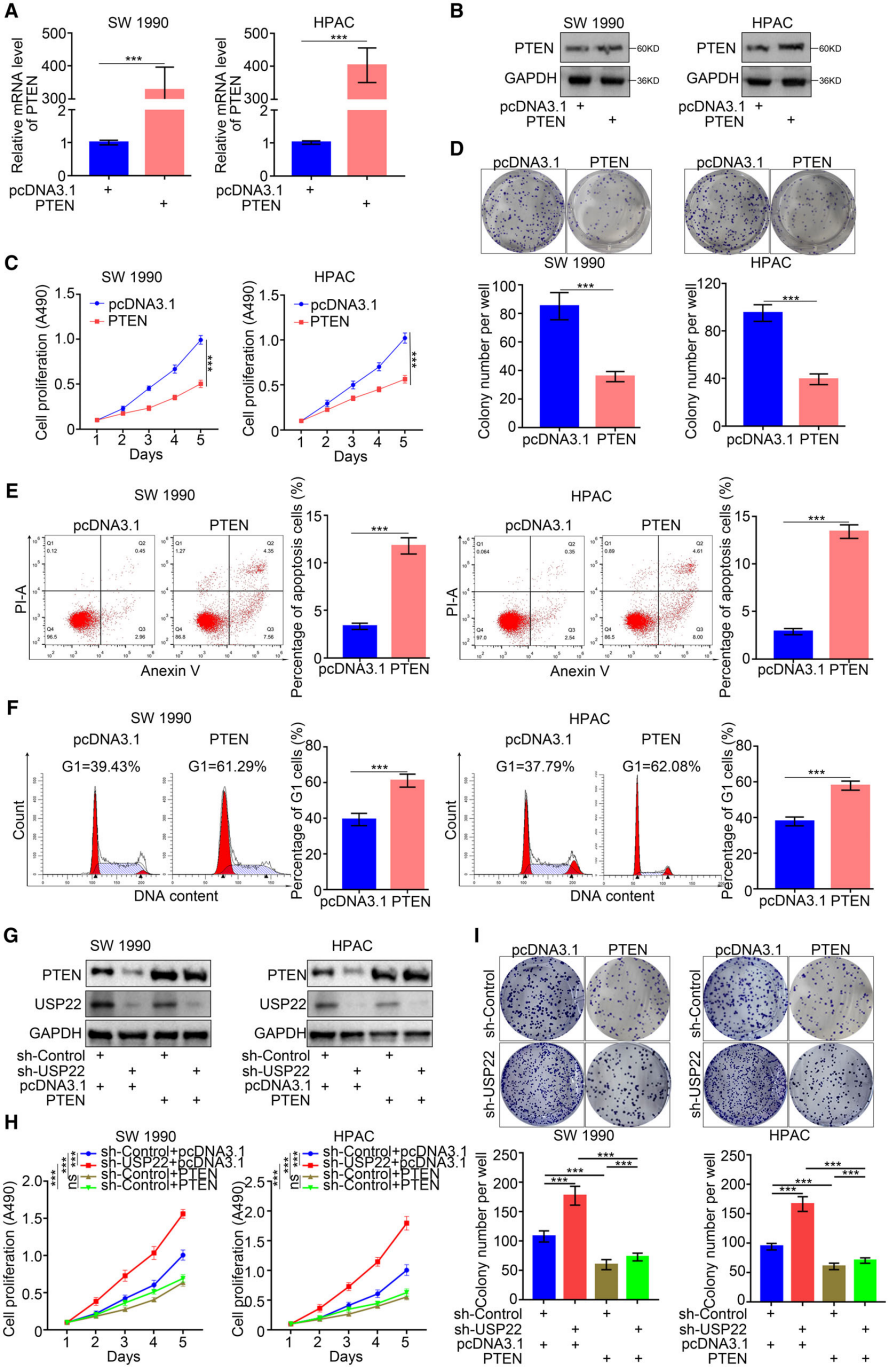
Restoring PTEN expression inhibited the progression of pancreatic cancer. (A,B) RT‐PCR (a) and western blot analysis (b) of PTEN expression in SW 1990 and HPAC cells infected with or without PTEN plasmid. GAPDH served as an internal reference. Data presented as the mean ± SD of three independent experiments. Statistical analyses were performed with one‐way ANOVA followed by Tukey's multiple comparison's tests. ***P < 0.001. (C,D) SW 1990 and HPAC cells infected with or without PTEN plasmid were harvested for MTS assay (c) and colony formation assay (d) after forty‐eight hours culturing. Each bar represents the mean ± SD of three independent experiments. Statistical analyses were performed with one‐way ANOVA followed by Tukey's multiple comparison's tests. ***P < 0.001. (E,F) SW 1990 and HPAC cells infected with or without PTEN plasmid were harvested for flow cytometry assays of apoptosis (E) and cell cycle (F) after forty‐eight hours culturing. Each bar represents the mean ± SD of three independent experiments. Statistical analyses were performed with one‐way ANOVA followed by Tukey's multiple comparison's tests. ***P < 0.001. (G) Western blot analysis of PTEN and USP22 expression in SW 1990 and HPAC cells infected with or without sh‐USP22 and infected with or without PTEN plasmid. GAPDH served as an internal reference and repeated for three replicates. (H,I) SW 1990 and HPAC cells infected with or without sh‐USP22 and infected with or without PTEN plasmid were harvested for MTS assay (H) and colony formation assay (I) after 48 h culturing. Each bar represents the mean ± SD of three independent experiments. Statistical analyses were performed with one‐way ANOVA followed by Tukey's multiple comparison's tests. ***P < 0.001. GAPDH: Glyceraldehyde‐3‐phosphate dehydrogenase.

As mentioned above, the PTEN protein level was regulated by USP22. However, PTEN reportedly can inhibit pancreatic cancer progression. Here, we verified the antitumor effects of PTEN in pancreatic cancer cells by establishing PTEN‐overexpressing SW 1990 and HPAC cell lines (Fig. 2A,B). MTS and colony formation assays demonstrated that PTEN overexpression led to the inhibition of pancreatic cancer cell proliferation (Fig. 2C,D). Additionally, the Annexin V/PI and cell cycle assays showed that PTEN significantly induced cell apoptosis and cell cycle arrest in pancreatic cancer (Fig. 2E,F). Furthermore, to determine that USP22 regulated the progression of pancreatic cancer by regulation PTEN expression, pancreatic cancer cells with or without USP22 silencing were infected with pcDNA3.1 or PTEN plasmids (Fig. 2G). The MTS assay and colony forming assays showed that USP22 silencing significantly inhibited the cell proliferation ability of pancreatic cancer cells, which could be reversed by PTEN overexpression (Fig. 2H,I). Therefore, our results indicate that the restoration of PTEN expression inhibited the progression of pancreatic cancer.

### 3.3 PTEN upregulated p21 expression in pancreatic cancer

**Fig. 3:**
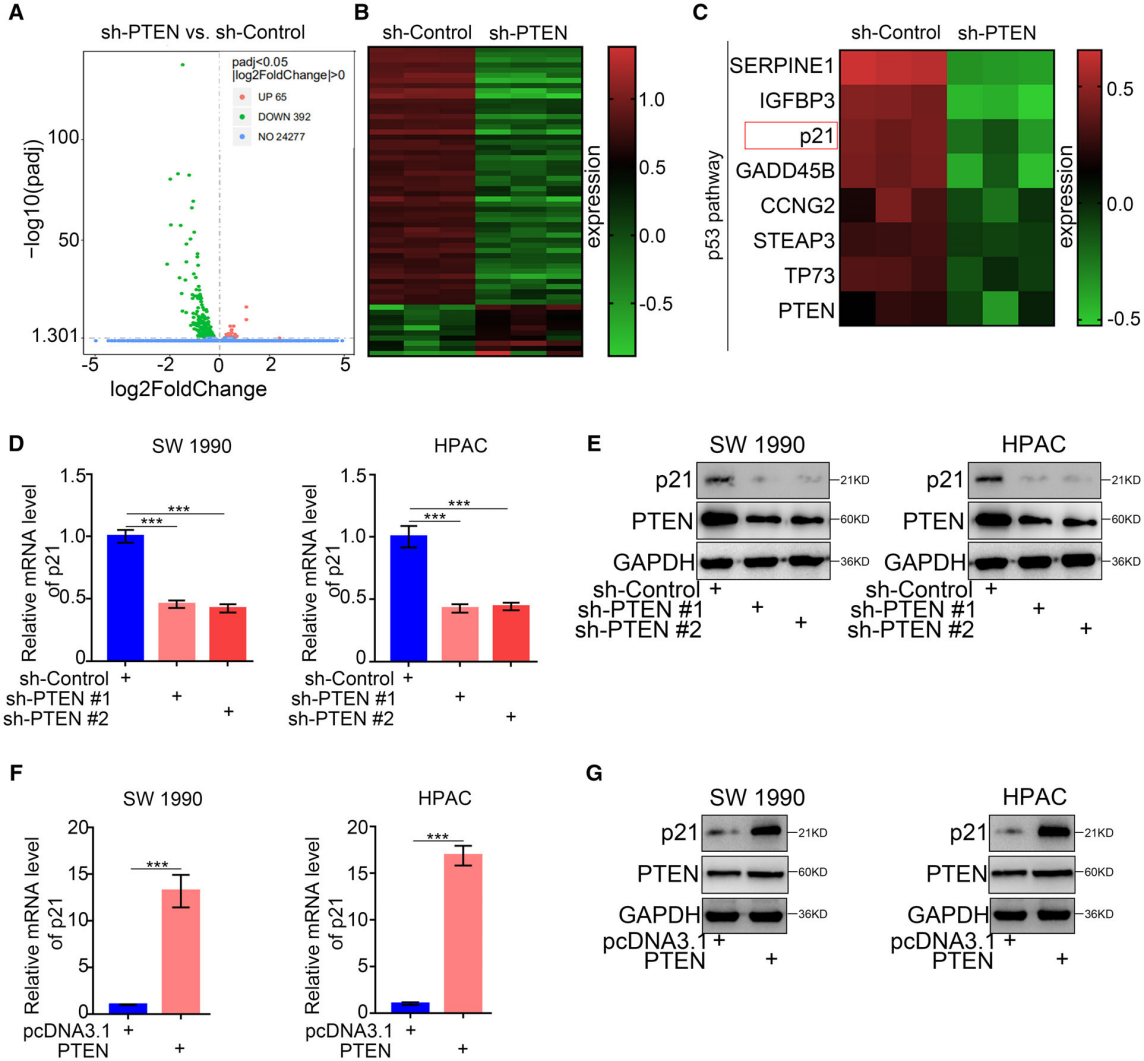
PTEN upregulated the expression of p21 in pancreatic cancer. (A,B) Volcano plot (A) and heatmap (B) to show the differential expressed genes of SW 1990 cells infected with sh‐Control or sh‐PTEN (*n* = 3). The green points represented the downregulated genes, while red points represented the upregulated genes. (C) Heatmap to show a subset of PTEN knockdown regulated genes participated in p53 pathway of SW 1990 cells (*n* = 3). (D,E) Forty‐eight hours postinfection, SW 1990 and HPAC cells infected with sh‐Control or sh‐PTENs were harvested for RT‐PCR analysis (d) and western blotting analysis (e). GAPDH served as an internal reference and repeated for three replicates. Data are shown as means ± SD (*n* = 3). Statistical analyses were performed with one‐way ANOVA followed by Tukey's multiple comparison's tests. ***P < 0.001. (F,G) Forty‐eight hours postinfection, SW 1990 and HPAC cells infected with pcDNA3.1 or PTEN plasmids were harvested for RT‐PCR analysis (F) and western blotting analysis (G). GAPDH served as an internal reference and repeated for three replicates. Data are shown as means ± SD (*n* = 3). Statistical analyses were performed with one‐way ANOVA followed by Tukey's multiple comparison's tests. ***P < 0.001. p21: Cyclin‐dependent kinase inhibitor 1A, also symboled as CDKN1A; GAPDH: Glyceraldehyde‐3‐phosphate dehydrogenase.

Because PTEN is critical for inhibiting pancreatic cancer progression, we employed RNA‐seq analysis to identify the underlying mechanism by which PTEN‐deubiquitinated USP22 regulates carcinogenesis. Our results identified 65 upregulated and 392 downregulated genes after the overexpression of PTEN in SW 1990 cells (Fig. 3A,B). Pathway analysis of the differentially expressed genes indicated that the p53 pathway was significantly regulated in pancreatic cancer cells that demonstrated PTEN inhibition. Notably, p21 expression was also downregulated by PTEN inhibition (Fig. 3C). Consistent with the findings of RNA‐seq analysis, PTEN silencing by two independent shRNAs led to decreased p21 expression in pancreatic cancer cells (Fig. 3D,E). Furthermore, p21 expression was upregulated at both the mRNA and protein levels in PTEN‐overexpressing cancer cells (Fig. 3F,G). These results indicate that PTEN transcriptionally regulates p21 expression in pancreatic cancer cells.

### 3.4 USP22 induced p21 expression via PTEN in pancreatic cancer

**Fig. 4:**
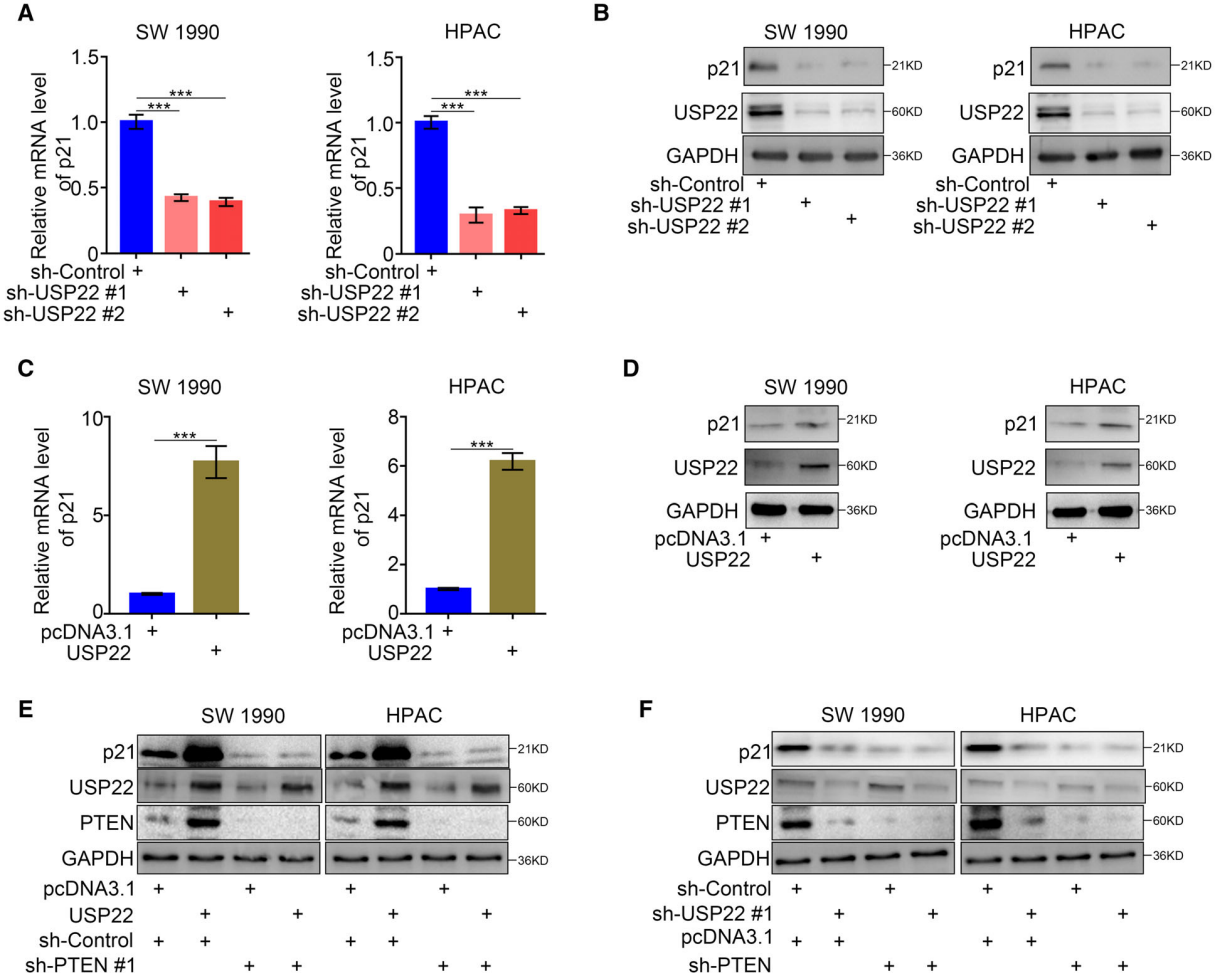
USP22 induced the p21 expression via PTEN in pancreatic cancer. (A,B) Forty‐eight hours postinfection, SW 1990 and HPAC cells infected with sh‐Control or sh‐USP22s were harvested for RT‐PCR analysis (A) and western blotting analysis (B). GAPDH served as an internal reference and repeated for three replicates. Data are shown as means ± SD (*n* = 3). Statistical analyses were performed with one‐way ANOVA followed by Tukey's multiple comparison's tests. ns, not significant; ***P < 0.001. (C,D) Forty‐eight hours postinfection, SW 1990 and HPAC cells infected with pcDNA3.1 or USP22 plasmids were harvested for RT‐PCR analysis (C) and western blotting analysis (D). GAPDH served as an internal reference and repeated for three replicates. Data are shown as means ± SD (*n* = 3). Statistical analyses were performed with one‐way ANOVA followed by Tukey's multiple comparison's tests. ns, not significant; ***P < 0.001. (E) Forty‐eight hours postinfection, SW 1990 and HPAC cells infected with or without USP22 plasmids and infected with or without sh‐PTEN were harvested for western blotting analysis. GAPDH served as an internal reference and repeated for three replicates. (F) Forty‐eight hours postinfection, SW 1990 and HPAC cells infected with or without sh‐USP22 and infected with or without sh‐PTEN were harvested for western blotting analysis. GAPDH served as an internal reference and repeated for three replicates. p21: Cyclin‐dependent kinase inhibitor 1A, also symboled as CDKN1A; GAPDH: Glyceraldehyde‐3‐phosphate dehydrogenase.

Because we demonstrated that USP22 deubiquitinated PTEN and inhibited the degradation of PTEN, we hypothesized that USP22 induced p21 expression via PTEN in pancreatic cancer. Our qRT‐PCR and western blotting assays demonstrated that USP22 silencing significantly inhibited p21 expression (Fig. 4A,B), while USP22 overexpression significantly promoted p21 expression (Fig. 4C,D). Furthermore, PTEN silencing reversed the upregulation of p21 induced by USP22 overexpression (Fig. 4E) and covered the downregulation of p21 induced by PTEN silencing (Fig. 4F). Taken together, our results indicated that USP22 induced p21 expression by deubiquitinating PTEN in pancreatic cancer.

### 3.5 USP22 upregulated p21 expression via PTEN‐MDM2‐p53 signaling in pancreatic cancer

**Fig. 5:**
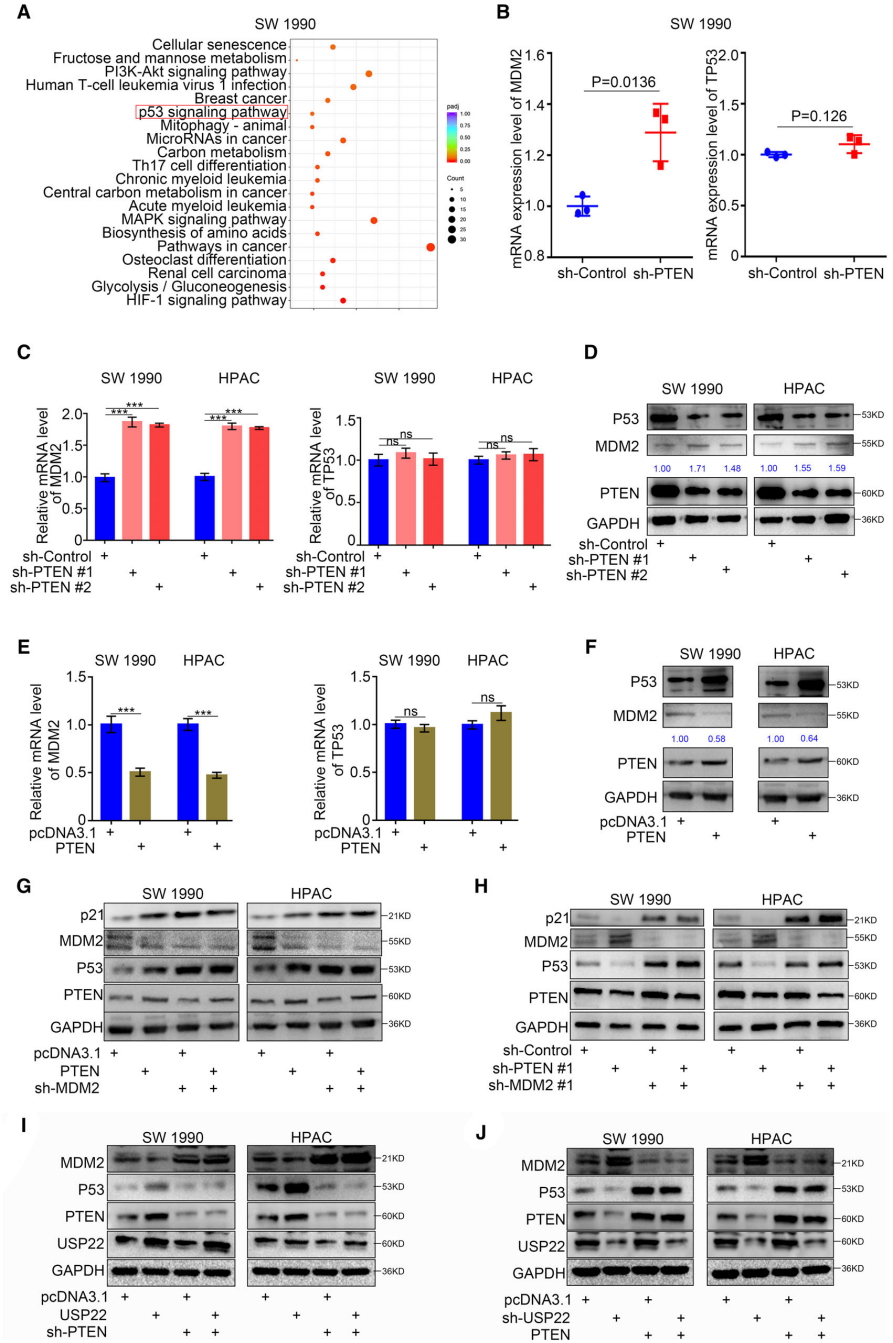
USP22 upregulated the expression of p21 via PTEN‐MDM2‐p53 signaling pathway in pancreatic cancer. (A) Bubble chart to show the KEGG enriched pathways of SW 1990 cells infected with sh‐PTEN. (B) Dot chart to show the mRNA expression level of p53 and MDM2 genes in SW 1990 cells infected with sh‐Control or sh‐PTEN. Data are shown as means ± SD (*n* = 3, *P* = 0.0136 and *P* = 0.126, respectively). Statistical analyses were performed with one‐way ANOVA followed by Tukey's multiple comparison's tests. (C,D) Forty‐eight hours postinfection, SW 1990 and HPAC cells infected with sh‐Control or sh‐PTENs were harvested for RT‐PCR analysis (C) and western blotting analysis (D). GAPDH served as an internal reference and repeated for three replicates. Data are shown as means ± SD (*n* = 3). Statistical analyses were performed with one‐way ANOVA followed by Tukey's multiple comparison's tests. ns, not significant; ***P < 0.001. (E,F) Forty‐eight hours postinfection, SW 1990 and HPAC cells infected with pcDNA3.1 or PTEN plasmids were harvested for RT‐PCR analysis (E) and western blotting analysis (F). GAPDH served as an internal reference and repeated for three replicates. Data are shown as means ± SD (*n* = 3). Statistical analyses were performed with one‐way ANOVA followed by Tukey's multiple comparison's tests. ns, not significant; ***P < 0.001. (G) Forty‐eight hours postinfection, SW 1990 and HPAC cells infected with or without PTEN plasmids and infected with or without sh‐MDM2 were harvested for western blotting analysis. GAPDH served as an internal reference and repeated for three replicates. (H) Forty‐eight hours postinfection, SW 1990 and HPAC cells infected with or without sh‐PTEN and infected with or without sh‐MDM2 were harvested for western blotting analysis. GAPDH served as an internal reference and repeated for three replicates. (I) Forty‐eight hours postinfection, SW 1990 and HPAC cells infected with or without USP22 plasmids and infected with or without sh‐PTEN were harvested for western blotting analysis. GAPDH served as an internal reference and repeated for three replicates. (J) Forty‐eight hours postinfection, SW 1990 and HPAC cells infected with or without sh‐USP22 and infected with or without sh‐PTEN were harvested for western blotting analysis. GAPDH served as an internal reference and repeated for three replicates. p21: Cyclin‐dependent kinase inhibitor 1A, also symboled as CDKN1A; GAPDH: Glyceraldehyde‐3‐phosphate dehydrogenase.

PTEN reportedly controls the expression and function of MDM2 by antagonizing the PI3K pathway and by negatively regulating the P1 promoter of the MDM2 gene [24]. MDM2 is a primary cellular inhibitor of p53; MDM2 inhibits p53 function by multiple mechanisms involving direct interactions [25]. Thus, we presumed that PTEN could regulate p21 expression via MDM2‐p53 signaling. Re‐analysis of the RNA‐seq data revealed that PTEN silencing significantly upregulated the expression of MDM2, but did not influence the mRNA expression of p53 (Fig. 5A,B). Furthermore, qRT‐PCR and western blotting assays verified that PTEN silencing significantly upregulated both the mRNA and protein expression levels of MDM2 (Fig. 5C,D), while PTEN overexpression significantly downregulated the mRNA and protein expression levels of MDM2 (Fig. 5E,F). Although PTEN did not influence the mRNA expression level of p53, PTEN silencing significantly downregulated the protein expression level of p53 (Fig. 5C,D), whereas PTEN overexpression significantly upregulated the protein expression level of p53 (Fig. 5E,F). Furthermore, MDM2 silencing covered the upregulation of p53 and p21 induced by PTEN overexpression (Fig. 5G) and reversed the downregulation of p53 and p21 induced by PTEN inhibition (Fig. 5H). Taken together, our results indicated that PTEN upregulated p21 expression via MDM2‐p53 signaling in pancreatic cancer.

Because PTEN regulated p21 expression via MDM2‐p53 signaling and USP22 induced the deubiquitination of PTEN in pancreatic cancer, we suspected that USP22 could also induce the MDM2‐p53 signaling pathway via PTEN in pancreatic cancer. We found that USP22 overexpression significantly inhibited the MDM2 expression and induced the protein expression of p53; these changes could be reversed by PTEN inhibition (Fig. 5I). Furthermore, USP22 silencing significantly induced the MDM2 expression and inhibited the protein expression of p53; these changes could be reversed by PTEN overexpression (Fig. 5J). Thus, our findings indicated that USP22 upregulated p21 expression via PTEN‐MDM2‐p53 signaling in pancreatic cancer.

### 3.6 MDM2 inhibitor enhanced the antipancreatic cancer effects of USP22 overexpression

**Fig. 6:**
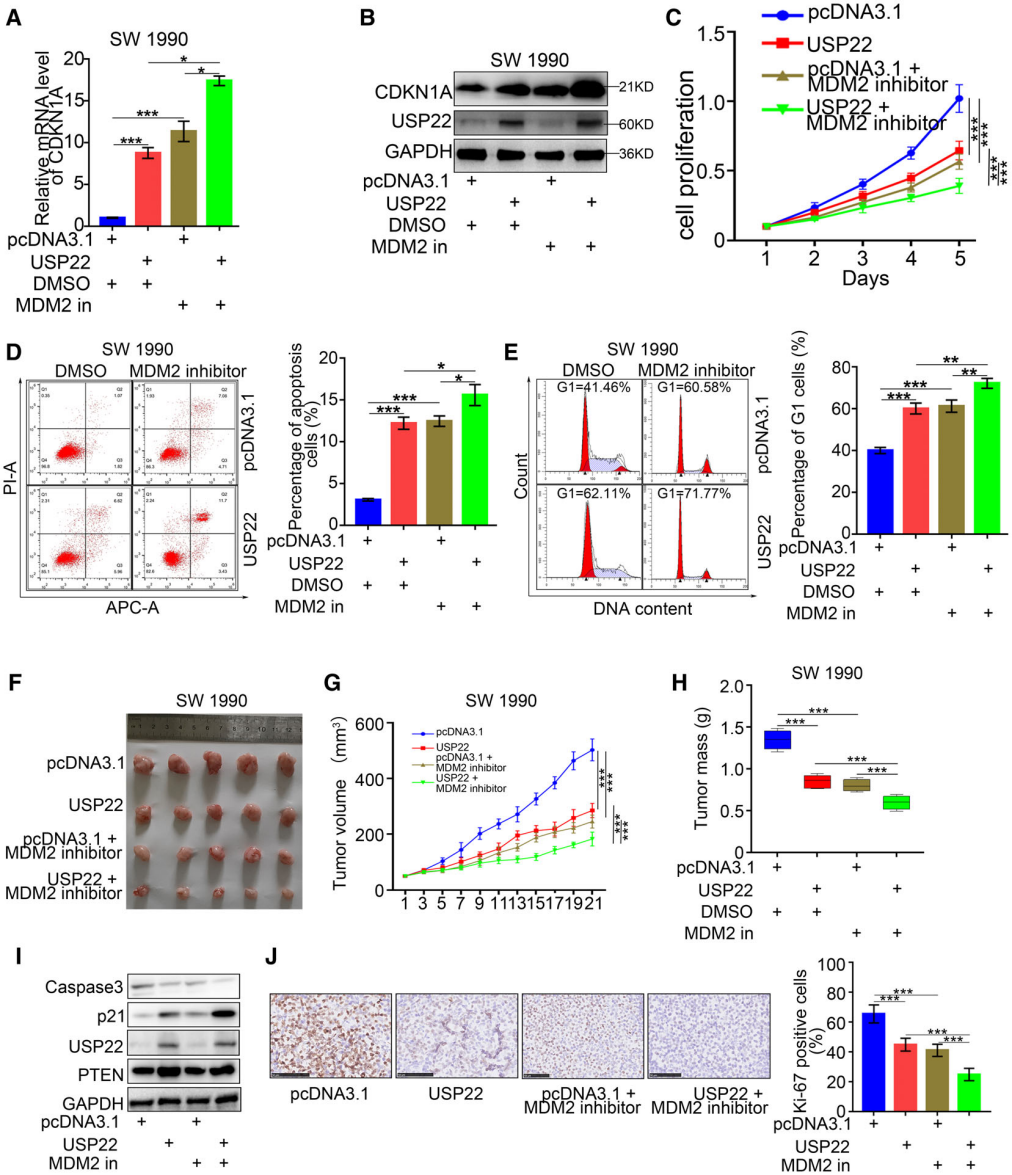
MDM2 inhibitor enhanced the antipancreatic cancer effects of USP22 overexpression. (A,B) RT‐PCR (A) and western Blot (B) analysis were used to detect the expression of p21 in SW 1990 cells. GAPDH served as an internal reference and repeated for three replicates. Statistical analyses were performed with one‐way ANOVA followed by Tukey's multiple comparison's tests. ns, not significant; *P < 0.05; ***P < 0.001. (C–E) SW 1990 cells infected with or without USP22 plasmid and treated with or without MDM2 inhibitor were harvested for MTS (C), apoptosis (D), and cell cycle assays (E). Each bar represents the mean ± SD of three independent experiments. Statistical analyses were performed with one‐way ANOVA followed by Sidak's multiple comparison's tests. *P < 0.05; **P < 0.01 ***P < 0.001. (F–H) SW 1990 cells infected with or without USP22 plasmid were subcutaneously injected into nude mice and with or without MDM2 inhibitor. The tumors were harvested and photographed (F) on day 21. Data for tumor volume (G) and tumor mass (H) are shown as the mean ± SD (*n* = 5). Statistical analyses were performed with two‐way ANOVA followed by Sidak's multiple comparison's tests. ***P < 0.001. (I) Mouse tumors were harvested for western blot analysis to detect the expression of p21 and caspase 3 (to determine the apoptosis level) in the tumors. GAPDH served as an internal reference and repeated for three replicates. (J) Mouse tumor sections were stained with Ki‐67 antibody to determine cell proliferation level. Data are shown as means ± SD (*n* = 5). The scale bar indicated 50um. Statistical analyses were performed with one‐way ANOVA followed by Tukey's multiple comparison's tests. ***P < 0.001. p21: Cyclin‐dependent kinase inhibitor 1A, also symboled as CDKN1A; GAPDH: Glyceraldehyde‐3‐phosphate dehydrogenase.

Nutlin‐3a, an MDM2 inhibitor, inhibits MDM2‐p53 interactions and stabilizes the p53 protein, thus inducing cell autophagy and apoptosis [26]. Because USP22 upregulated p21 expression via PTEN‐MDM2‐p53 signaling in pancreatic cancer, we hypothesized that the combination of USP22 overexpression and MDM2 inhibitor treatment would have an enhanced antipancreatic cancer effect. qRT‐PCR and western blotting analysis showed the enhanced effects of USP22 overexpression and MDM2 inhibitor treatment in the induction of p21 expression (Fig. 6A,B). In addition, MTS, apoptosis, and cell cycle assays indicated the enhanced effects of USP22 overexpression and MDM2 inhibitor treatment in the inhibition of proliferation, and the induction of apoptosis and cell cycle arrest, in pancreatic cancer cells *in vitro* (Fig. 6C,D). Furthermore, subcutaneous injection of normal USP22‐expressing or USP22‐overexpressing SW 1990 cells into the left flank of nude mice for a xenograft assay, followed by mouse treatment with or without MDM2 inhibitor, revealed that USP22 overexpression and MDM2 inhibitor treatment could both slow tumor growth; the combined treatment group showed additional inhibition of tumor growth (Fig. 6F–H). Consistently, the western blot assay showed that the expression of p21 was also upregulated in the tumors of both USP22 overexpression and MDM2 inhibitor treatment group, especially in the combined treatment group (Fig. 6I). Furthermore, the proliferation and apoptosis levels were also inhibited in the tumors of both USP22 overexpression and MDM2 inhibitor treatment group, especially in the combined treatment group (Fig. 6I,J). Overall, these data showed that MDM2 inhibitor enhanced the antipancreatic cancer effects of USP22 overexpression *in vitro* and *in vivo*.

### 3.7 PTEN upregulated p21 expression by interacting with ANKHD1 in pancreatic cancer

**Fig. 7:**
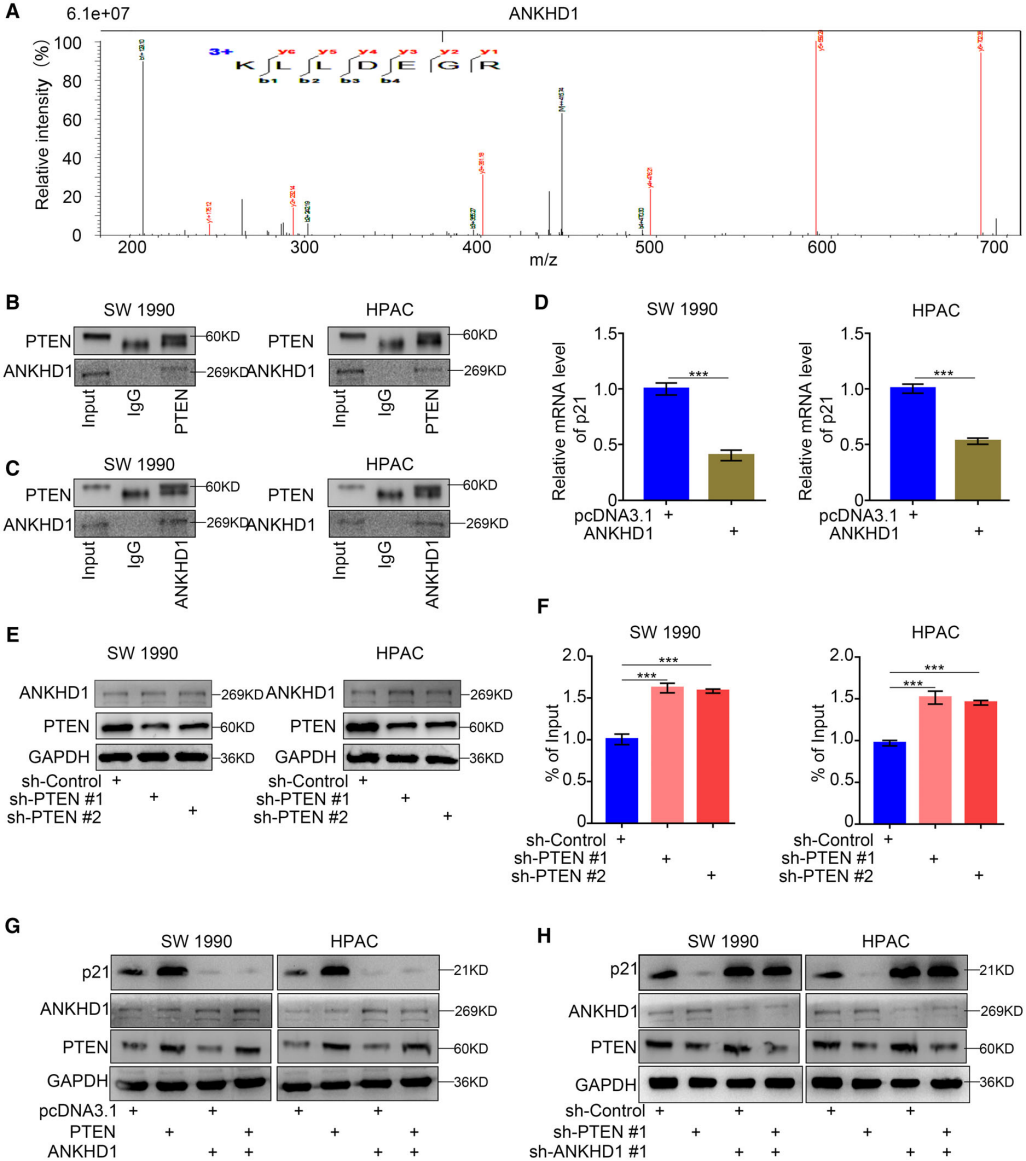
PTEN upregulated the expression of p21 via interacting with ANKHD1 in pancreatic cance. (A,B) LC‐MS/MS identified an interaction between PTEN and ANKHD1 (A) by detecting a peptide of ANKHD1 (B, *n* = 1). (C) Coimmunoprecipitation showed the interaction between PTEN and ANKHD1, which repeated for three replicates. (D) RT‐PCR analysis to show the protein expression levels of specific genes. Data are shown as means ± SD (*n* = 3). GAPDH served as an internal reference. Statistical analyses were performed with one‐way ANOVA followed by Tukey's multiple comparison's tests. ***P < 0.001. (E,F) RT‐PCR analysis (E) and western blotting analysis (F) to show the protein expression levels of specific genes. Data are shown as means ± SD (*n* = 3). GAPDH served as an internal reference and repeated for three replicates. Statistical analyses were performed with one‐way ANOVA followed by Tukey's multiple comparison's tests. ***P < 0.001. (G,H) Western blotting analysis to show the protein expression levels of specific genes. GAPDH served as an internal reference and repeated for three replicates. p21: Cyclin‐dependent kinase inhibitor 1A, also symboled as CDKN1A; GAPDH: Glyceraldehyde‐3‐phosphate dehydrogenase.

As mentioned above, PTEN upregulated p21 expression in pancreatic cancer, in a manner that depended on the MDM2‐p53 signaling pathway. However, the involvement of other mechanisms by which PTEN upregulates p21 expression has been unclear. To address this uncertainty, we re‐analyzed the LC‐MS/MS data and identified ANKHD1 as a potential PTEN‐interacting protein (Fig. 7A,B); this finding was verified by immunoprecipitation assays (Figs 7C and S2). ANKHD1 reportedly downregulated p21 promoter activity and repressed p21 expression [27]. In our study, we found that the overexpression of ANKHD1 significantly inhibited p21 expression in pancreatic cancer (Fig. 7D). Additionally, PTEN did not regulate the expression of ANKHD1, but significantly regulated the degree of ANKHD1 binding to the p21 promoter (Fig. 7E,F). Furthermore, ANKHD1 overexpression reversed the upregulation of p21 induced by PTEN overexpression (Fig. 7G), while ANKHD1 silencing reversed the downregulation of p21 induced by PTEN inhibition (Fig. 7H). Therefore, the regulation of p21 by PTEN was dependent on ANKHD1 in pancreatic cancer cells.

## 4 Discussion

PTEN is a tumor suppressor gene that is inhibited in various human malignancies. Normal expression of PTEN induces growth suppression by promoting cell cycle arrest, while PTEN downregulation in pancreatic carcinoma is presumably an important genetic event that induces aggressive malignancy (e.g., proliferation and invasion). Novel therapeutic molecular approaches have promising positive effects on pancreatic cancer, involving enhanced expression of PTEN and limited proliferation of cancer cells [28]. Ubiquitination modification is an important method for PTM of PTEN in malignant cancers. A previous study showed that TRIM37 mediated chemoresistance and stemness maintenance in pancreatic cancer cells by ubiquitination of PTEN [29]. Similarly, TG2 promoted degradation of PTEN by the ubiquitin‐proteasomal pathway in pancreatic cancer [30]. However, the deubiquitination modification of PTEN in pancreatic cancer, which inhibits the degradation of PTEN, has been unclear. In the present study, we identified a novel deubiquitination‐modifying enzyme of PTEN, USP22, which induced the deubiquitination of PTEN and inhibited its degradation. We also searched the GEPIA database, which showed a significant correlation between the mRNA expression levels of USP22 and PTEN in TCGA patient samples (Fig. S3). Besides, USP22 was not a differential expressed gene in PTEN silenced pancreatic cancer cells (Fig. S4). Therefore, there might be an unknown regulation mechanism at the mRNA level between USP22 and PTEN, which should explore in the further research. Furthermore, the overexpression of USP22 could inhibit the progression of pancreatic cancer by inducing PTEN expression. Thus, restoration of USP22 expression is a novel therapeutic strategy to induce PTEN expression in pancreatic cancer.

PTEN is a frequently mutated tumor suppressor, and over two thirds of PTEN missense variants currently do not have a clear interpretation of significance, which means that the contribution of these variants to cancer still is unclear [31]. There are multiple mechanisms of PTEN inactivation in cancer, including loss of PTEN's lipid and protein phosphatase activities and reduced protein stability [32]. Therefore, the PTEN could divide into two types, including functional PTEN and unfunctional PTEN in cancer cells. In our study, we just researched the functional PTEN, which was the basis of functional assays that was to say the PTEN stabled by USP22 was a functional PTEN.

Upon exposure to DNA damage or other stressors, p21 expression was activated by tumor suppressor p53 [33]. As mentioned above, previous studies showed that PTEN negatively regulated the P1 promoter of the MDM2 gene, a primary cellular inhibitor of p53, through a direct interaction, thus antagonizing the PI3K pathway [24, 25]. In our study, we verified that PTEN induced p21 expression via MDM2‐p53 signaling in pancreatic cancer. Our study also identified a novel mechanism by which PTEN induced p21 expression in pancreatic cancer cells; this could contribute to the interaction with ANKHD1 and inhibit binding to the p21 promoter of ANKHD1. Since the development and application of MDM2 inhibitors in cancer therapy, the target PTEN‐MDM2‐p53‐p21 pathway has become increasingly important in clinical therapy for pancreatic cancer. Furthermore, the exploration of ANKHD1 inhibitor treatment could provide novel therapy strategies for pancreatic cancer, especially in combination with MDM2 inhibitor treatment, which should be verified by further research.

USP22, the deubiquitinating subunit of the SAGA transcriptional cofactor complex, is a member of an 11‐gene ‘death‐from‐cancer’ signature [22]. The functions of USP22 in malignant cancers have not been fully elucidated. Many previous studies have reported the cancer‐promoting potential of USP22, including the induction of cell proliferation and DNA repair [34], activation of c‐Myc/NAMPT/SIRT1‐dependent FOXO1 and YAP signaling [35], and deubiquitination of CD274 to suppress anticancer immunity [36]. In contrast, USP22 can exert tumor‐suppressive functions in CRC, such that its loss increases CRC burden by modulating mTOR activity [22]. In our study, we identified a novel mechanism underlying the USP22 anticancer effect, which could contribute to the onset of deubiquitination and inhibit the degradation of PTEN. Furthermore, USP22 could induce p21 expression via PTEN‐MDM2‐p53 and PTEN‐ANKHD1 signaling pathways. Therefore, a clearer understanding of methods to enhance anticancer effects and inhibit the cancer‐promoting potential of USP22 would provide new treatment options for pancreatic cancer.

MDM2 functions as an effective p53 antagonist in cells with wild‐type p53. The MDM2 protein binds to the p53 protein directly through their amino termini and inhibits p53 function through direct ubiquitination of p53 with its E3 ligase activity, thereby promoting proteasomal degradation of p53 [37, 38, 39]. Many drugs/compounds have been developed to facilitate reactivation of the p53 gene; these include mechanisms such as inhibiting MDM2 interaction with p53, using MDM2 antagonism, and inhibiting E3 ubiquitination of p53. Because USP22 could induce p21 expression via PTEN‐MDM2‐p53 signaling, we hypothesized that MDM2 inhibitor treatment enhanced the antipancreatic cancer effect of USP22 overexpression, which had been verified by combined approach of MDM2 inhibitor treatment and USP22 overexpression *in vivo* and *in vitro*. Therefore, MDM2 comprises a novel therapeutic target for pancreatic cancer.

ANKHD1 (ankyrin repeat and K‐homology domain containing 1) is a large protein characterized by the presence of a K‐homology domain, which could bind to RNA or ssDNA, and is found in proteins associated with transcriptional and translational regulation [40, 41]. ANKHD1 were widely expressed in normal tissues and cancer cell lines at different levels, and the aberrant expression of ANKHD1 has been reported in some types of cancer [42]. Previous studies had shown the evidence of ANKHD1 in the malignant phenotype of cancer cells, including promoting cancer cell proliferation, migration, invasion, and tumorigenesis [42], by positively regulating of YAP1 [43, 44], JAK/STAT [45], and STMN1 [46, 47], and negatively regulating p21 [48]. In our study, we demonstrated that PTEN could induce p21 expression via interacting with ANKHD1 and enhancing the binding degree of ANKHD1 to the p21 promoter in pancreatic cancer cells. Therefore, ANKHD1 might also be a novel therapeutic target of pancreatic cancer.

## 5 Conclusions

In conclusion, our study identified USP22 as a novel deubiquitination‐modifying enzyme of PTEN. By inducing deubiquitination and inhibiting the degradation of PTEN, USP22 could also induce p21 expression in pancreatic cancer. Besides, MDM2 inhibitor enhanced the antipancreatic cancer effect of USP22 overexpression. In addition to regulating MDM2‐p53 signaling, our study also identified a novel mechanism by which PTEN induced p21 expression through an interaction with ANKHD1 and inhibition of binding to the p21 promoter of ANKHD1. Taken together, these findings indicate that ANKHD1 and MDM2 might be a novel therapeutic target in pancreatic cancer.

## Conflict of interest

The authors declare no conflict of interest.

## Author contributions

DR and YS performed the experiments; DL collected the data; XJ and HW wrote the paper and analyzed the data. All authors read and approved the final manuscript.

### Peer Review

The peer review history for this article is available at https://publons.com/publon/10.1002/1878‐0261.13137.

## Supporting information


**Fig. S1**. The IC50 curve of Nutlin‐3a in pancreatic cancer cells.
**Fig. S2**. Coimmunoprecipitation showed the interaction between PTEN and USP22, and between PTEN and ANKHD1 in 293T cells.
**Fig. S3**. RNA‐seq assay showed the mRNA expression level of USP22 in PTEN silenced SW 1990 cells.
**Fig. S4**. GEPIA database was searched for the correlation between PTEN and USP22 mRNA expression.
**Table S1**. The primer sequences for RT‐qPCR.
**Table S2**. The shRNA sequences.
**Table S3**. The primer sequences for ChIP‐qPCR.

## Data Availability

Please contact the corresponding author (Xin Jin, jinxinunion@hust.edu.cn; Heshui Wu, heshuiwu@hust.edu.cn) for data requests.
