# Supplementary material for: USP22‐mediated deubiquitination of PTEN inhibits pancreatic cancer progression by inducing p21 expression
Source: Mol Oncol. 2021 Nov 16;16(5):1200–17. doi: 10.1002/1878-0261.13137 (PMC8895442; doi:10.1002/1878-0261.13137)
Supplement: Supplementary file 1 — Fig. S1. The IC50 curve of Nutlin‐3a in pancreatic cancer cells. Fig. S2. Coimmunoprecipitation showed the interaction between PTEN and USP22, and between PTEN and ANKHD1 in 293T cells. Fig. S3. RNA‐seq assay showed the mRNA expression level of USP22 in PTEN silenced SW 1990 cells. Fig. S4. GEPIA database was searched for the correlation between PTEN and USP22 mRNA expression. Table S1. The primer sequences for RT‐qPCR. Table S2. The shRNA sequences. Table S3. The primer sequences for ChIP‐qPCR. [file MOL2-16-1200-s001.docx]

**Supplementary Data**

**Supplementary figure 1.** The IC50 curve of Nutlin-3a in pancreatic cancer cells.


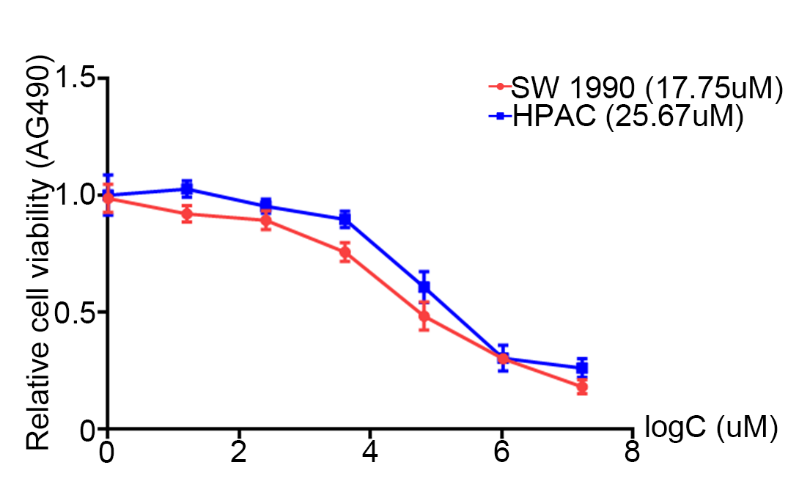


**Supplementary figure 2.** Coimmunoprecipitation showed the interaction between PTEN and USP22, and between PTEN and ANKHD1 in 293T cells, which repeated for three replicates.


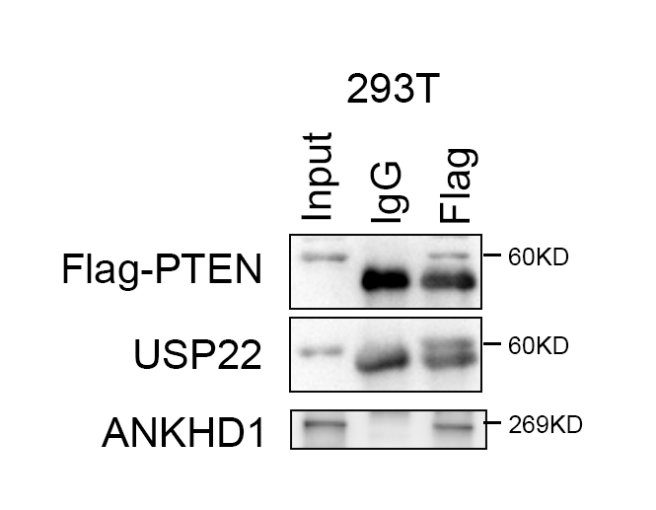


**Supplementary figure 3.** RNA-seq assay showed the mRNA expression level of USP22 in PTEN silenced SW 1990 cells (n=3). Statistical analyses were performed with one-way ANOVA followed by Tukey's multiple comparison's tests. ns, not significant.


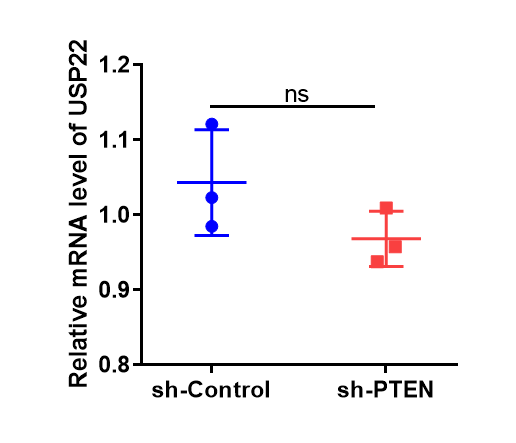


**Supplementary figure 4.** GEPIA database was searched for the correlation between PTEN and USP22 mRNA expression.


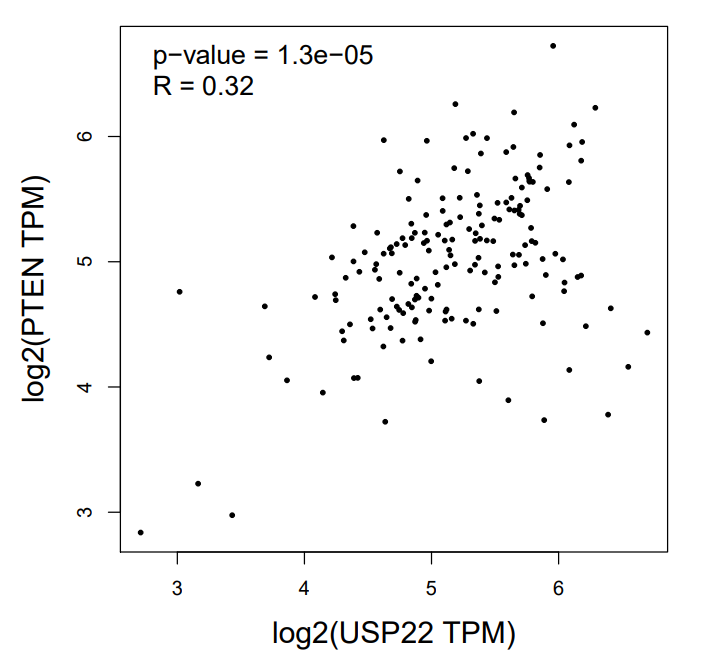


**Supplementary table 1. The primer sequences for RT-qPCR.**

| Gene | Forward primer (**5**′ - 3′) | Reverse primer (**5**′ - 3′) |
| --- | --- | --- |
| **GAPDH** | ATGACAATGAATACGGCTACAGCA | GCAGCGAACTTTATTGATGGTATT |
| **PTEN** | TGGATTCGACTTAGACTTGACCT | GGTGGGTTATGGTCTTCAAAAGG |
| **p21** | TGTCCGTCAGAACCCATGC | AAAGTCGAAGTTCCATCGCTC |
| **MDM2** | GAATCATCGGACTCAGGTACATC | TCTGTCTCACTAATTGCTCTCCT |
| **p53** | CAGCACATGACGGAGGTTGT | TCATCCAAATACTCCACACGC |

**Supplementary table 2. The shRNA sequences.**

| **Sh-PTEN #1** | CCGGCTAGAACTTATCAAACCCTTTCTCGAGAAAGGGTTTGATAAGTTCTAGTTTTTG |
| --- | --- |
| **Sh-PTEN #2** | CCGGAGGCGCTATGTGTATTATTATCTCGAGATAATAATACACATAGCGCCTTTTTTG |
| **Sh-ANKHD1 #1** | CCGGGCAGTCTAGCAGAAGCTTGTTCTCGAGAACAAGCTTCTGCTAGACTGCTTTTTTG |
| **Sh-ANKHD1 #2** | CCGGGCACTACTCTTAGCACAAGGACTCGAGTCCTTGTGCTAAGAGTAGTGCTTTTTTG |
| **Sh-MDM2 #1** | CCGGATTATCTGGTGAACGACAAAGCTCGAGCTTTGTCGTTCACCAGATAATTTTTTG |
| **Sh-MDM2 #2** | CCGGTAGTATAATTGACCTACTTTGCTCGAGCAAAGTAGGTCAATTATACTATTTTTG |
| **Sh-USP22 #1** | CCGGAGCTACCAGGAGTCCACAAAGCTCGAGCTTTGTGGACTCCTGGTAGCTTTTTTG |
| **Sh-USP22 #2** | CCGGCGAAGGGTACTTGCTGTTCTACTCGAGTAGAACAGCAAGTACCCTTCGTTTTTG |

**Supplementary table 3. The primer sequences for ChIP-qPCR.**

| Gene | Forward primer (**5**′ - 3′) | Reverse primer (**5**′ - 3′) |
| --- | --- | --- |
| **p21** | CCTCTTCGGGTGGAC | CCGTTTTCGACCCTGAGAG |
